# Concomitant Unilateral/Bilateral Temporomandibular Joint Reconstruction and Maxillomandibular Advancement for Temporomandibular Joint Pathologies and Obstructive Sleep Apnea: Technical Note and Case Report

**DOI:** 10.3390/jcm14051719

**Published:** 2025-03-04

**Authors:** Jean-Pierre T.F. Ho, Ning Zhou, Cornelis Klop, Nadeem R. Saeed, Jan de Lange

**Affiliations:** 1 Amsterdam UMC Location University of Amsterdam, Department of Oral and Maxillofacial Surgery, University of Amsterdam, Meibergdreef 9, 1105 AZ Amsterdam, The Netherlands; 2 Academic Centre for Dentistry Amsterdam (ACTA), University of Amsterdam and Vrije Universiteit Amsterdam, 1081 LA Amsterdam, The Netherlands; 3 Department of Oral and Maxillofacial Surgery, Northwest Clinics, 1815 JD Alkmaar, The Netherlands; 4 Department of Oral and Maxillofacial Surgery, Great Ormond Street Hospital, London WC1N 3JH, UK

**Keywords:** obstructive sleep apnea, ankylosis, temporomandibular joint reconstruction, maxillomandibular advancement, Le Fort I osteotomy

## Abstract

**Background:** Patients with a triad of severe temporomandibular joint (TMJ) pathologies, obstructive sleep apnea (OSA), and dentofacial deformities often experience significant functional and aesthetic impairments. A combination of total TMJ reconstruction and maxillomandibular advancement (MMA) has emerged as a promising treatment option, which can address the complexity of these conditions simultaneously. **Methods:** This paper presents a practical protocol for TMJ reconstruction using patient-specific alloplastic total joint prosthesis in conjunction with additional splintless osteotomies. This approach integrates the recent advancements in virtual surgical planning (VSP), custom TMJ prostheses, and three-dimensional (3D) custom osteotomy guide and implant manufacturing, allowing for precise anatomical correction and enhanced treatment outcomes. Three patients were treated with the present protocol. Postoperative assessments mainly included maximum inter-incisal opening, apnea–hypopnea index (AHI), and patient satisfaction with facial aesthetics. **Results:** All surgeries were performed without complications. The follow-up period ranged from 7 to 12 months. For the two patients with TMJ ankylosis, the postoperative maximum inter-incisal opening (MIO) increased from 3–5 to 35 mm and from 12 to 32 mm, respectively. Additionally, all three cases demonstrated that the protocol could significantly decrease AHI (with improvements of 57.5, 49, and 66.4 events/h, respectively) and achieve satisfactory aesthetics. **Conclusions:** These findings suggest that this protocol is a viable option for addressing complex cases involving severe TMJ pathologies, OSA, and dentofacial deformities. Future studies with larger cohorts and long-term follow-up are needed to further validate these findings.

## 1. Introduction

Temporomandibular joint (TMJ) pathologies requiring total joint reconstruction are often complex, involving conditions that compromise joint structure and function to a degree that non-surgical interventions are insufficient [1,2]. These pathologies can arise from a range of etiologies, including degenerative diseases like osteoarthritis, systemic inflammatory conditions such as rheumatoid arthritis, congenital malformations, trauma, infections, and ankylosis [2,3,4]. Recently, TMJ reconstruction with an alloplastic total joint prosthesis has gained more interest, showing satisfactory results [3,5,6]. With the introduction of three-dimensional (3D) imaging modalities, computer-aided design and manufacturing (CAD/CAM), and 3D printing, total joint prosthesis systems have introduced the patient-specific—also known as custom-made—prosthesis, in addition to the stock non-patient-specific prosthesis [7,8].

Patients affected by TMJ pathologies may experience various symptoms, such as chronic pain, restricted jaw mobility, malocclusion, facial asymmetry, and airway obstruction [9,10]. In patients with TMJ pathologies, the degree of airway compromise can vary and range from minor complaints to the presence of obstructive sleep apnea (OSA) [10,11]. OSA is a sleep-related breathing disorder, which is characterized by recurrent periods of partial and/or complete upper airway collapses during sleep, leading to multiple episodes of breathing cessation and arousals [12]. The gold standard therapy for OSA to date is continuous positive air pressure (CPAP) [13,14]. However, the main limitation of CPAP is the lack of adherence among some patients [15,16]. For those who are CPAP-intolerant, maxillomandibular advancement (MMA) is considered a highly effective alternative [13]. The standard approach includes a Le Fort I osteotomy for maxillary repositioning and a bilateral sagittal split osteotomy (BSSO) for mandibular advancement [13]. By simultaneous advancement of the maxilla and mandible, MMA can significantly enlarge the upper airway and stiffen the pharyngeal soft tissues [17].

The current gold standard for orthognathic surgery is to virtually plan the surgical procedures in 3D and transfer the surgical planning with 3D-printed intraoperative surgical splints [18]. Over the years, a novel method to transfer the planning has been proposed, in which patient-specific osteotomy guides and fixation implants are used for positioning the osteotomized jaws, without the need for intraoperative surgical splints [19]. The splintless technique has been suggested to enhance surgical accuracy and reduce operation time [20].

In cases with TMJ pathologies, OSA, and dentofacial deformities, different single or combined treatment options have been suggested, such as mandibular distraction, gap arthroplasty, TMJ reconstruction with autologous bone or total joint alloplastic prostheses, and maxilla osteotomy [10,11,21,22,23]. TMJ reconstruction with total joint alloplastic prostheses combined with a maxillary osteotomy—which can be considered a modified MMA—has been reported to address TMJ pathologies and OSA simultaneously [24,25]. This approach provides both functional and airway benefits by correcting skeletal discrepancies, optimizing occlusion, and improving airway patency. However, to the best of the authors’ knowledge, the application of the splintless technique in the modified MMA has not been reported. The rationale for the splintless technique is that it can improve accuracy in occlusal alignment and condylar seating, thereby facilitating both early functional recovery and long-term stability while optimizing airway outcomes.

The aim of this technical note was to present a protocol for TMJ reconstruction using patient-specific total joint alloplastic implants combined with a splintless Le Fort I osteotomy in patients with severe TMJ pathologies, OSA, and dentofacial deformities. We also assess the outcomes of this procedure in three patients, focusing on both functional and polysomnographic results.

## 2. Technical Note

### 2.1. Clinical Protocol

During the preoperative clinical investigation, the facial profile and dimensions—including comprehensive soft tissue and skeletal cephalometric analyses, chin and nose position, facial asymmetries, and the occlusal relationship—are assessed. Patient photographs and 3D stereophotogrammetry are also obtained. After dental cast models are acquired, they are digitized by CBCT scanning, converted to stereolithography (STL) files, and incorporated into computed tomography (CT) scans for virtual surgical planning (VSP). The CT imaging protocol can be found in Table 1. The severity of OSA is objectively assessed with an overnight polysomnography (PSG).

### 2.2. Virtual Surgical Planning and Computer-Aided Manufacturing Workflow

**Figure 1:**
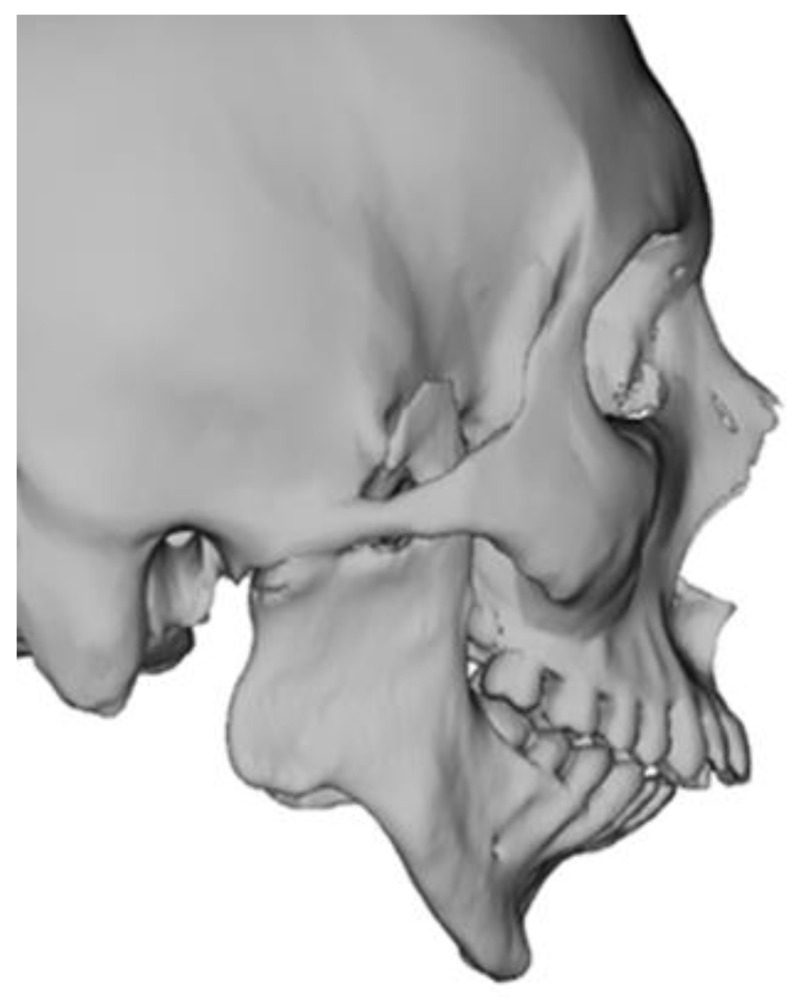
Lateral view of natural head position.

To virtually plan the surgery, CT data are exported in Digital Imaging and Communications in Medicine (DICOM) format and subsequently imported into the VSP^®^ System software (3D Systems Inc., Littleton, CO, USA). As the first step, a 3D virtual hard-tissue model of the patient is reconstructed; when available, STL files of the dental cast models are fused with the hard-tissue model to enhance anatomical accuracy. The 3D virtual hard-tissue model is aligned in the natural head position (NHP) based on clinical assessment and patient photographs (Figure 1) [26].

**Figure 2:**
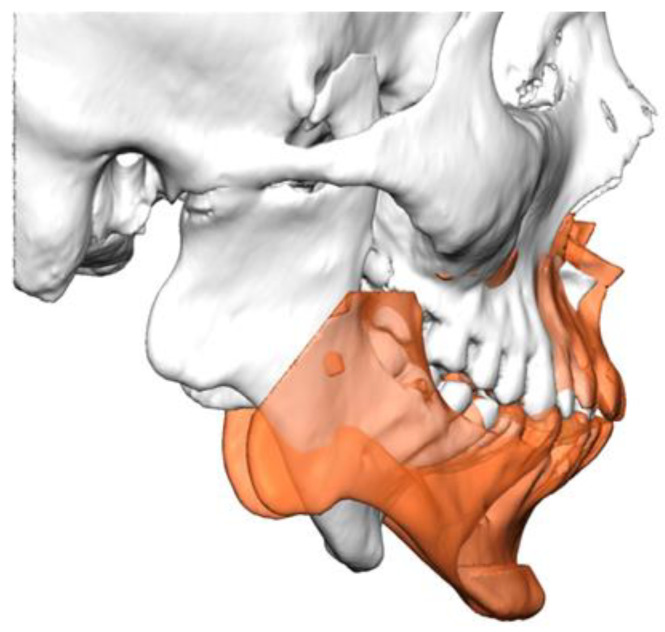
Virtual surgical planning of the final position (orange) of the maxillomandibular complex.

The maxilla is virtually osteotomized according to a Le Fort I or segmental Le Fort I. In cases where mandibular asymmetry or unilateral TMJ pathology is present, a unilateral sagittal split osteotomy is planned on the contralateral side of the mandible. The maxillomandibular complex is then virtually advanced and rotated counterclockwise to the desired final position. During the VSP phase, the maximal advancement is determined to achieve the desired reduction in apnea and hypopnea events while also ensuring an aesthetically acceptable outcome and considering potential soft tissue restrictions (Figure 2).

Next, the resection of the condylar head, coronoid process, and glenoid fossa are virtually planned.

**Figure 3:**
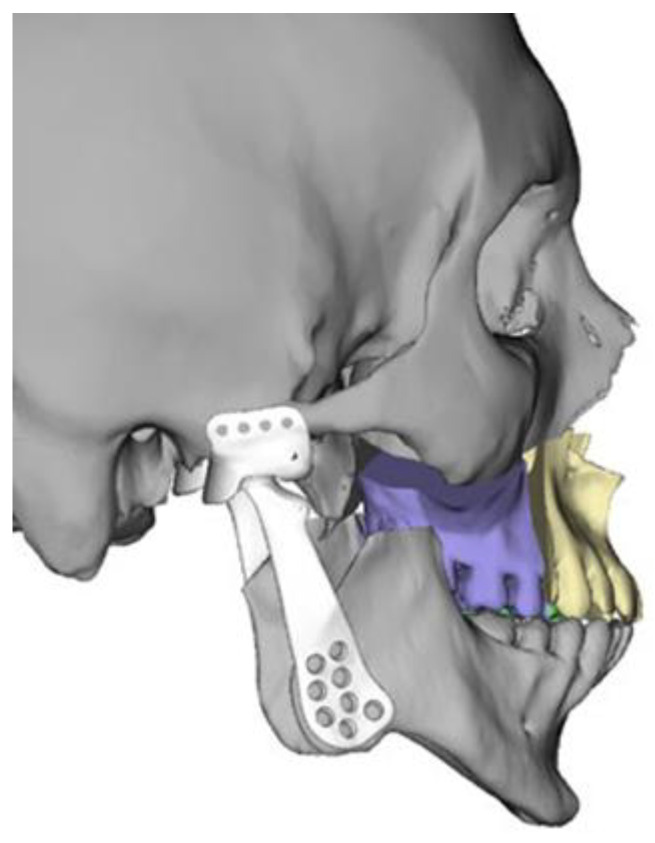
Design of the patient-specific total joint prosthesis (white).

After planning the resections and final position of the maxillomandibular complex with the assistance of VSP^®^ System software (3D Systems Inc., Littleton, CO, USA), patient-specific total joint implants (bilateral or unilateral) and resection guides for the mandible and glenoid fossa are designed and fabricated (Custom Patient Matched TMJ Implants, Zimmer Biomet CMF & Thoracic, Jacksonville, FL, USA) (Figure 3) [27].

The resection guides incorporate identifiable and clear anatomical landmarks to facilitate accurate resection and prosthesis placement. Additionally, they include the final screw hole positions for fixation of the custom total joint implants.

**Figure 4:**
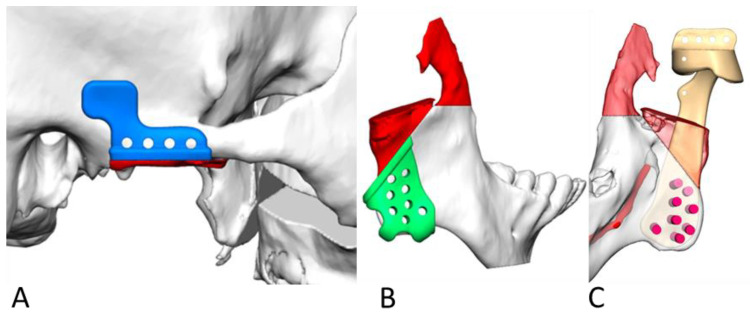
Virtual design of the resection guides include the final screw hole positions for fixation for the glenoid fossa (blue) (**A**) and ramus (green) (**B**) for drilling and cutting. (**C**) Virtual design of the position of the prosthesis (gold), including a medium posterior lip and anterior suture holes design.

For the glenoid fossa implant in cases of large advancements, a 3 to 5 mm posterior lip on the glenoid fossae is recommended to reduce the risk of dislocation. Additionally, the posterior lip margin should be positioned at least 3 mm from the external auditory canal to prevent ear canal impingement (Figure 4A–C) [27].

Suture holes in both the mandibular and fossa components can be incorporated to allow the placement of a non-resorbable suture, helping to prevent immediate condylar inferior displacement—also known as sag.

If unilateral sagittal split osteotomy is planned, a patient-specific implant (PSI) for fixation, along with an osteotomy guide embedded with pre-planned screw hole positions of the fixation PSI, are designed with Blender software version 2.8 (Blender Foundation, Amsterdam, NH, The Netherlands) and Meshmixer 3.5 (Autodesk inc., San Rafael, CA, USA) and 3D printed in titanium (KLS Martin Group, Tuttlingen, Germany).

**Figure 5:**
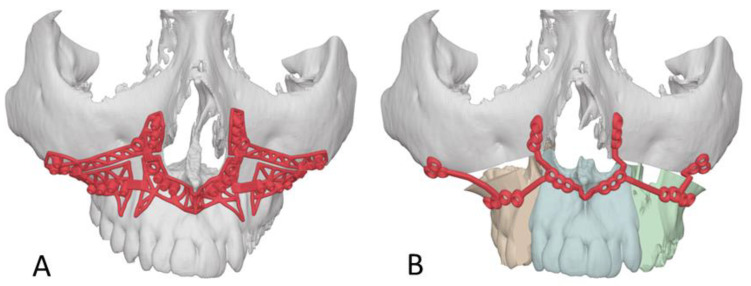
Virtual design of the osteotomy cutting guide (red) (**A**) and patient-specific implant (red) (**B**) for the Le Fort I osteotomy.

Based on the planned final position of the maxilla, an osteotomy guide incorporating pre-planned screw hole positions for the PSI and a PSI for fixation, are designed and 3D-printed (Figure 5A,B) [28]. The design and manufacturing process of the Le Fort I guide is similar to the previously described process for the unilateral sagittal split osteotomy guide and PSI.

A mandible-first surgical protocol is commonly preferred for the procedure; however, it is a recommendation rather than a mandatory requirement. An intermediate splint is designed and fabricated to correctly position the mandible relative to the maxilla during intermaxillary fixation (IMF).

### 2.3. Surgical Technique

#### 2.3.1. Mandible

**Figure 6:**
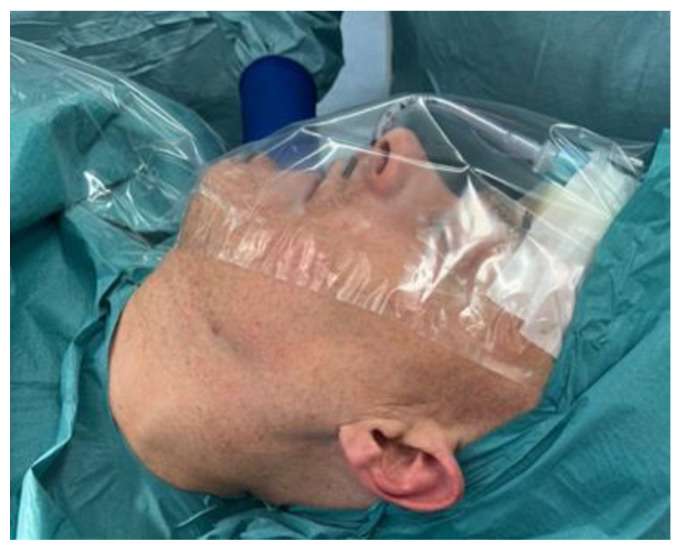
Nasotracheal intubation.

**Figure 7:**
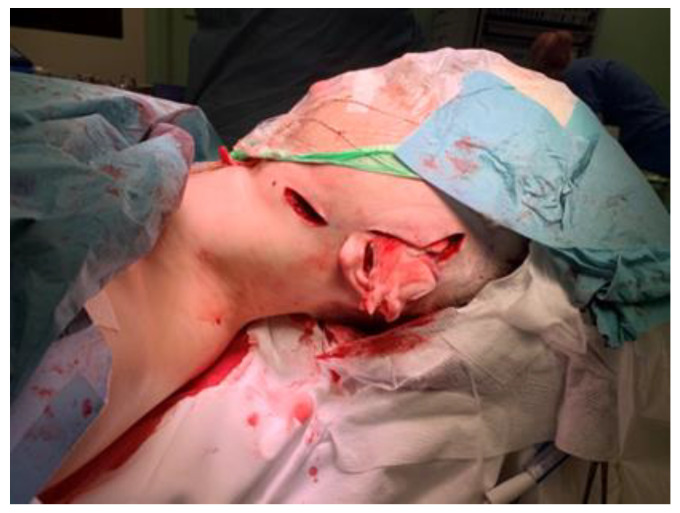
Preauricular and retromandibular incisions.

**Figure 8:**
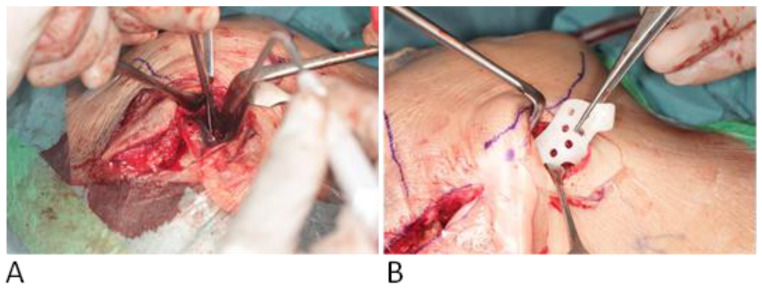
Intraoperative placement of the resection guides for the glenoid fossa (**A**) and iramus (**B**).

The patient is positioned in the supine position. General anesthesia is administered through nasotracheal intubation (Figure 6). Local anesthesia is then injected to help with hemostasis. In cases where bilateral TMJ reconstruction is performed, the bilateral TMJs are accessed via preauricular and retromandibular incisions (Figure 7). Care should be taken to position the retromandibular incisions to avoid overlapping the mandibular ramus component, especially in cases of severe rotation. Cutting guides are placed at the mandibular rami and glenoid fossae, and the final screw holes are pre-drilled. For temporary fixation of the cutting guides, shorter screws are used, in contrast to the longer screws used for the final fixation of the prosthesis. Condylectomy, coronoidectomy, and the resection of the glenoid fossa are then performed (Figure 8) [27].

If the orthodontic fixed appliance is not applied preoperatively, the SMARTLock Hybrid MMF bars (Stryker, Corp., Kalamazoo, MI, USA) are placed for IMF. Next, the maxilla and the mandibula are placed into maximal occlusion in IMF using the intermediate splint [29]. To prevent cross-contamination with intraoral bacteria, a separate set of instruments is used for the intraoral procedure, and the patient is re-draped before proceeding with the extraoral procedure.

**Figure 9:**
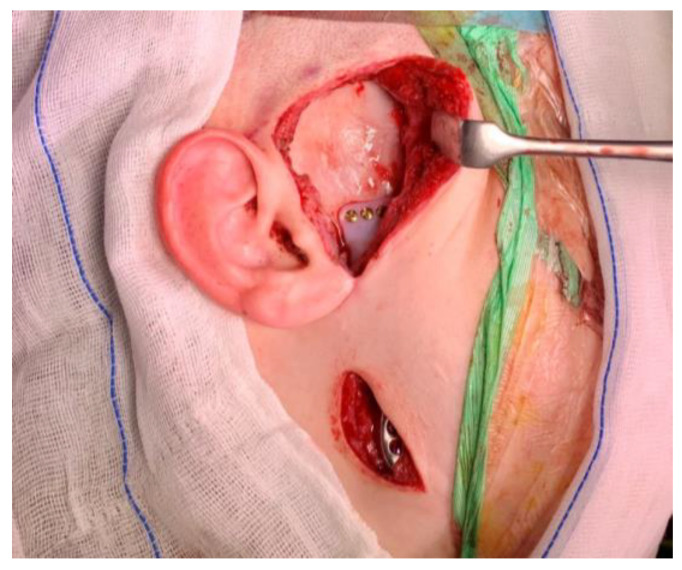
Placement of the glenoid fossa and mandibular ramus components of the prosthesis.

**Figure 10:**
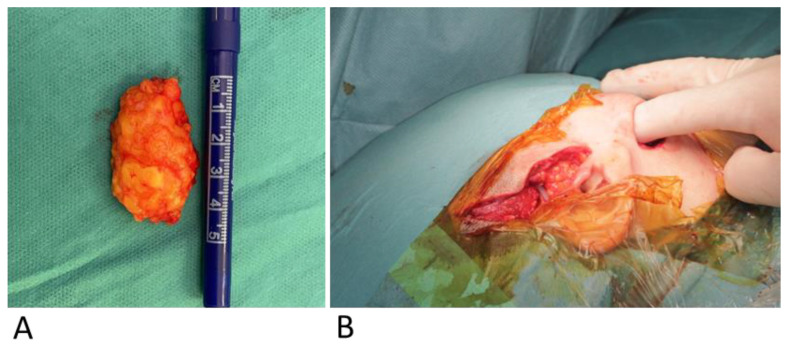
(**A**) Fat harvested from abdomen. (**B**) Fat grafted around the total joint prosthesis.

Proceeding with the extraoral procedure, the trial prosthesis is placed to verify the correct positioning of the final prosthesis. Once proper placement is confirmed, the final prosthesis is inserted. The glenoid fossa component is placed first, followed by the mandibular ramus component, with both components secured using the pre-drilled screw holes (Figure 9). Abdominal fat is harvested and grafted around the total joint implant to obturate the gap and prevent re-ankylosis and heterotopic bone formation (Figure 10B) [30]. When it is a bilateral TMJ reconstruction case, the same procedures are then repeated on the contralateral side. Before initiating intraoral procedures, a thorough inspection for any perforation from the extraoral joints should be conducted, and the most effective approach to achieve an airtight closure should be determined to prevent contamination. Subsequently, the incisions should be meticulously sutured.

**Figure 11:**
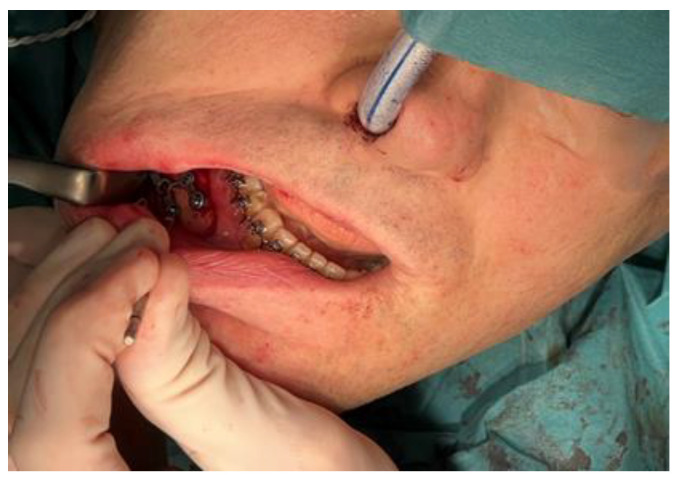
The fixation patient-specific implant for the sagittal split placed and fixated on the right side of the mandible.

When it is a unilateral TMJ reconstruction case, the unilateral sagittal split osteotomy on the contralateral side is then performed after the TMJ reconstruction. A mucosal incision is made in the retromolar area. Subperiosteal dissection is performed to expose the lateral aspect of the mandible, the anterior ramus, and the medial ramus above the inferior alveolar nerve. The customized cutting guide is then positioned, and screw holes are pre-drilled. Vertical and horizontal osteotomies are made with the piezotome, and the guide is then removed. After the osteotomy is completed using chisels, splitting forceps and an elevator are used to separate the bony segments. The inferior alveolar nerve is then identified. Next, the PSI is applied and secured with screws provided by the manufacturer (Figure 11). Additional procedures, such as genioplasty, may be performed when necessary.

#### 2.3.2. Maxilla

**Figure 12:**
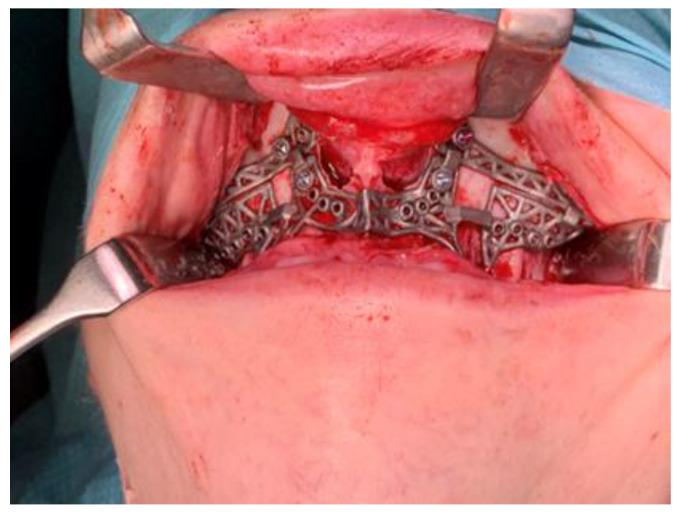
The Le Fort I osteotomy cutting guide positioned and temporarily fixated to the maxilla.

**Figure 13:**
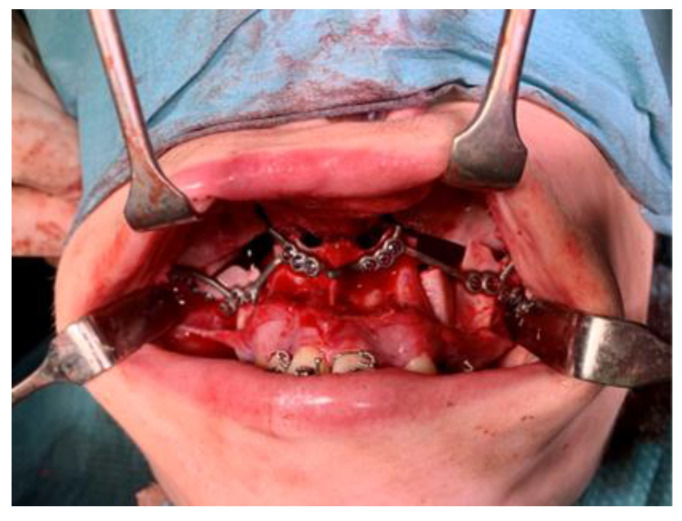
The fixation patient-specific implant for the maxilla placed and fixated in the planned position.

If present, the previously placed arch bars and screws are removed prior to Le Fort I osteotomy. A maxillary gingivobuccal incision is made from the canine regions using a minimally invasive approach. Subperiosteal dissection is performed to expose the anterior and lateral wall of the maxilla from the piriform rims to the pterygoid processes. The nasal mucosa is detached. Then, the patient-specific cutting guide is applied (Figure 12). Fixation holes are pre-drilled, followed by creating osteotomy cuts. The guide is then removed, and the osteotomy is extended with a saw. A subspinal osteotomy is performed with chisels, and a U-shaped or V-shaped osteotome is used to separate the nasal septum from the maxilla. Afterwards, the maxilla is down-fractured and mobilized. A segmental osteotomy is created with the piezotome as indicated. The fixation PSI for the maxilla is then placed and fixated with the provided screws into the pre-drilled holes, by which the maxilla is placed into the planned position (Figure 13).

Postoperatively, patients undergo a stringent postoperative rehabilitation regime—described by De Meurechy et al.—with the help of a dietitian, speech therapist, physical therapist, and dentist specialized in gnathology [31]. During the first 7 days postoperatively, patients are permitted only passive and active mouth opening and closing. After this period, they can begin using the Therabite system (Atos Medical, Malmö, Sweden) several times a day for less than 10 s per session, for a duration of one to three weeks. In the following weeks, patients are instructed to gradually increase the intensity and frequency of Therabite use.

#### 2.3.3. Outcome Evaluation

Evaluation CT scan is acquired within seven days postoperatively using the same scanning protocol as preoperatively. The scan is performed with the patient’s mouth closed in a relaxed jaw position, ensuring that the mandibular ramus component is properly seated in the glenoid fossa component of the TMJ prosthesis. To assess the effectiveness of the surgery on OSA, the patient will undergo a PSG at least 3 months postoperatively.

## 3. Case Report

### 3.1. Case 1

**Figure 14:**
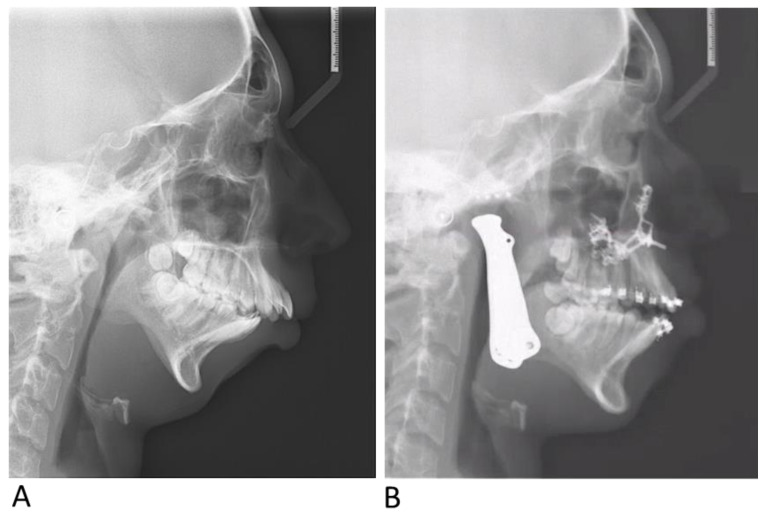
Preoperative (**A**) and postoperative (**B**) lateral cephalometric radiographs of case 1.

A 16-year-old male was referred to the Department of Oral and Maxillofacial Surgery due to severe limited mouth opening. The patient had a medical history of a TMJ infection at toddler age, which resulted in a bilateral TMJ ankylosis. The patient received bilateral gap arthroplasty at the age of 12 years but still experienced restricted function. The patient also suffered from severe OSA, for which he has been using CPAP for many years. Upon examination, the patient presented with a maximal inter-incisal opening (MIO) of 3–5 mm, severe retrognathia, disharmony of the facial aesthetic, Class II malocclusion, and a cavity in two molars. A CT scan confirmed residual bilateral TMJ ankylosis. PSG showed an apnea–hypopnea index (AHI) of 62.5 events/h. After a multidisciplinary discussion between an ENT surgeon, maxillofacial surgeon, orthodontist, and gnathology and restorative dentist, a treatment protocol was determined involving bilateral TMJ reconstruction with custom total joint alloplastic prosthesis and a splintless segmental Le Fort I osteotomy as described above. The surgery proceeded as planned, and the postoperative course was uneventful. Immediately following surgery, the patient experienced a marked improvement in symptoms, with the complete resolution of excessive daytime somnolence and, therefore, discontinued the use of CPAP (Figure 14A,B). A PSG 3 months postoperatively showed an AHI of 4.8 events/h. One year postoperatively, the patient exhibited an MIO of 35 mm and had satisfactory aesthetic and functional outcomes.

### 3.2. Case 2

**Figure 15:**
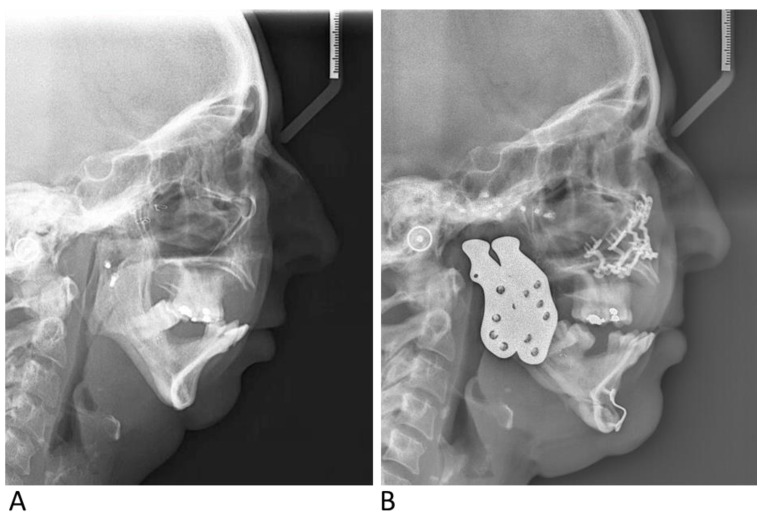
Preoperative (**A**) and postoperative (**B**) lateral cephalometric radiographs of case 2.

A 49-year-old female patient with a history of bilateral TMJ ankylosis resulting from facial trauma, for which she underwent gap arthroplasty with costochondral graft reconstruction 26 years earlier. The patient reported limited mouth opening, pain, severe OSA with CPAP intolerance, and unsatisfactory facial aesthetics. Clinical and radiographic examinations revealed residual bilateral TMJ ankylosis, a mutilated dentition with multiple missing teeth (extracted to allow for emergency intubation in case of airway compromise), a restricted inter-incisal opening of 12 mm, retrognathia, a Class II malocclusion, and disharmonious facial aesthetics. PSG confirmed a diagnosis of severe OSA, with an AHI of 50 events/h. Consensus was achieved during a multidisciplinary discussion to treat the patient according to the treatment protocol involving a bilateral TMJ reconstruction with custom total joint alloplastic prosthesis combined with a splintless Le Fort I osteotomy and a genioplasty. The surgery proceeded without complications (Figure 15A,B). Postoperatively, the patient was relieved from pain. A follow-up PSG three months later showed an AHI of 1 event/h. One year postoperatively, the patient demonstrated an MIO of 32 mm. She was also very satisfied with the aesthetic outcome.

### 3.3. Case 3

**Figure 16:**
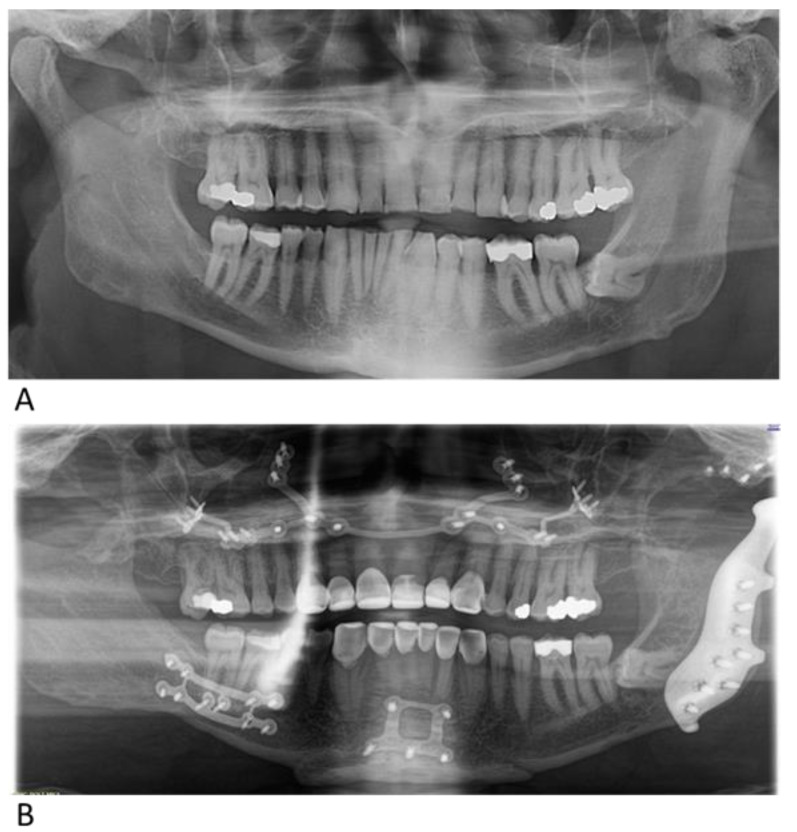
Preoperative (**A**) and postoperative (**B**) panoramic radiographs of case 3.

The patient is a 55-year-old male presented to our department with the chief complaints of progressive asymmetric growth of his face, complete open bite, and severe OSA (AHI = 77 events/h). The patient wished to restore occlusion and symmetry and seek a long-term solution for OSA. Upon clinical examination, there was a chin deviation of 12 mm to the right, audible sounds on the left TMJ when opening and closing the jaw, an open bite of 4 mm, and severe tooth wear. A CT scan showed osteochondroma of the left condyle with secondary subluxation of the left TMJ and asymmetry of the mandible. The preoperative phase involved orthodontic treatment. Surgical procedures that were performed included resection of the osteochondroma and reconstruction of the left TMJ with custom total joint alloplastic prosthesis, sagittal ramus split on the right side, Le Fort I osteotomy, and genioplasty. The postoperative course was uneventful (Figure 16A,B). Seven months after surgery, the patient showed facial symmetry and proper occlusion. His joint was functioning normally, with no signs of discomfort or restricted movement. Postoperative PSG revealed an AHI of 10.6 events/h.

## 4. Discussion

It is true that the triad of severe TMJ pathologies, OSA, and dentofacial deformities is uncommon, but patients with these conditions often experience serious physical and mental impacts, including, among others, functional impairments, facial disharmony, sleep-related symptoms, and, depending on the degree of disfigurement, psychosocial difficulties [32].

TMJ reconstruction with custom total joint alloplastic prosthesis has been well-documented in the management of severe TMJ pathologies such as TMJ ankylosis [8,27,33]. The application of a splintless protocol in MMA for severe OSA unrelated to TMJ pathologies has also been reported [13,28]. Although a few studies proposed the combination of total TMJ reconstruction and MMA in the treatment of TMJ pathologies and OSA, as far as authors are aware, no studies have combined splintless MMA with TMJ reconstruction [24,25]. Therefore, the aim of this paper was to present and evaluate the treatment protocol that integrates total TMJ reconstruction with patient-specific total joint alloplastic implants combined with a splintless Le Fort I osteotomy for patients presenting with TMJ pathologies, OSA, and dentofacial deformities. The ultimate goal of this combined approach was to restore TMJ function, improve airway patency, and achieve optimal dentofacial aesthetics and occlusion while minimizing surgical morbidity.

The treatment protocol demonstrated promising outcomes, showing a significant increase in MIO, reduction in the AHI, and improvement in facial aesthetics. All three patients were pleased with the treatment outcomes. This finding aligns with the study by Movahed et al., supporting the efficacy of total TMJ reconstruction in conjunction with MMA for addressing concomitant TMJ pathologies and OSA [25]. To confirm its treatment outcomes and long-term stability, applying this protocol in a larger study population with long-term follow-up is necessary in future research. Additionally, longitudinal studies assessing patient-reported outcomes, including quality of life, functional satisfaction, and psychosocial well-being, would be valuable in determining the overall impact of this surgical approach.

Patient safety is a critical aspect of surgical intervention, particularly in complex procedures such as TMJ reconstruction combined with a Le Fort I osteotomy. Due to the complexity of this combined approach, there may be concerns regarding potential challenges such as managing postoperative complications (e.g., nerve injury, TMJ prosthesis failure, and heterotopic bone formation) and maintaining occlusal stability [34]. In the three cases presented, the surgeries proceeded as planned, and the postoperative course was uneventful, demonstrating the safety of the protocol. It is well known that meticulous surgical planning and dedicated technique are paramount to reducing the risk of complications. Advancements in 3D imaging, CAD/CAM design, and custom manufacturing have allowed surgeons to anticipate and manage potential complications more effectively. In this protocol, the use of patient-specific cutting guides and fixation implants enhanced surgical precision and reduced the likelihood of intraoperative errors [20]. The use of custom-made TMJ prostheses further allowed safer and easier procedures, especially in complex cases, by improving anatomical fit and simplifying the surgical process [35]. Despite its advantages, this protocol is not without limitations. Firstly, the requirement for advanced technology may limit the accessibility of the present treatment protocol in resource-limited settings. The cost of patient-specific TMJ prostheses, as well as custom osteotomy guides and implants, may also be a barrier for some patients. Future research should be performed to assess the cost-effectiveness of this approach compared to separate procedures or staged approaches. Secondly, evaluating postoperative accuracy relative to VSP is considered challenging. Specifically, assessing the accuracy of the position of the glenoid fossa component was limited by its translucency on the CT scan. Additionally, the mandible component, as well as the tooth-bearing distal mandibular segment, could not be evaluated in 3D. Comparison of the pre- and postoperative mandibular positions relative to the cranial base is burdensome due to the inherent mobility of the mandible. Feasible and repeatable methods for objectively assessing the placement accuracy of both genoid fossae and mandibular rami components should be developed, which would enhance the reliability and reproducibility of surgical outcomes. Thirdly, patient selection remains a crucial consideration. This protocol is applied to patients with severe TMJ pathologies, OSA, and dentofacial deformities. Future studies are needed to establish standardized criteria for patient eligibility to optimize clinical outcomes.

## 5. Conclusions

Custom total joint alloplastic TMJ reconstruction combined with a splintless Le Fort I osteotomy can offer an effective approach for addressing TMJ pathologies, OSA, and dentofacial deformities. This protocol demonstrates improvements in mandibular function, airway obstruction, and facial aesthetics, enhancing patient quality of life. While initial outcomes are promising, further research with a large study population is necessary for broader validation.

## Figures and Tables

**Table 1:** The CT imaging protocol of the three patients.

**Manufacturer**	Siemens		
**Scanner model**	SOMATOM Force		
**Contrast use**	No		
**Reconstruction kernel**	Hr64 (bone kernel)		
**Field-of-view**	Full skull		
**Collimation width (mm)**	96 × 0.6		
**Matrix size**	512 × 512		
**Slice thickness (mm)**	1.0		
**Slice increment (mm)**	1.0		
**kVp**	120	120	150
**X-ray tube current (mA)**	82	61	211
**Exposure time (ms)**	1000	1000	1000
**Pitch factor**	0.55	0.55	0.8
**Pixel spacing (mm)**	0.46 × 0.46	0.48 × 0.48	0.48 × 0.48

## Data Availability

The data presented in this study are available on request from the corresponding author.
